# Comprehensive transcriptome analysis and functional characterization of PR-5 for its involvement in tomato *Sw-*7 resistance to tomato spotted wilt tospovirus

**DOI:** 10.1038/s41598-019-44100-x

**Published:** 2019-05-21

**Authors:** Chellappan Padmanabhan, Qiyue Ma, Reza Shekasteband, Kevin S. Stewart, Samuel F. Hutton, John W. Scott, Zhangjun Fei, Kai-Shu Ling

**Affiliations:** 1 USDA-Agricultural Research Service, U.S. Vegetable Laboratory, Charleston, South Carolina USA; 2 Boyce Thompson Institute, Cornell University, Ithaca, New York USA; 3 University of Florida, IFAS, Gulf Coast Research and Education Center, Wimauma, FL USA; 4 USDA-Agricultural Research Service, Robert W. Holley Center for Agriculture and Health, Ithaca, New York USA

**Keywords:** Molecular engineering in plants, Biotic

## Abstract

Tomato spotted wilt tospovirus (TSWV), one of the most important plant viruses, causes yield losses to many crops including tomato. The current disease management for TSWV is based mainly on breeding tomato cultivars containing the *Sw-*5 locus. Unfortunately, several *Sw-5* resistance-breaking strains of TSWV have been identified. *Sw-7* is an alternative locus conferring resistance to a broad range of TSWV strains. In an effort to uncover gene networks that are associated with the *Sw-7* resistance, we performed a comparative transcriptome profiling and gene expression analysis between a nearly-isogenic *Sw-7* line and its susceptible recurrent parent (Fla. 8059) upon infection by TSWV. A total of 1,244 differentially expressed genes were identified throughout a disease progression process involving networks of host resistance genes, RNA silencing/antiviral defense genes, and crucial transcriptional and translational regulators. Notable induced genes in *Sw-7* include those involved in callose accumulation, lignin deposition, proteolysis process, transcriptional activation/repression, and phosphorylation. Finally, we investigated potential involvement of *PR-5* in the *Sw-7* resistance. Interestingly, PR-5 overexpressed plants conferred enhanced resistance, resulting in delay in virus accumulation and symptom expression. These findings will facilitate breeding and genetic engineering efforts to incorporate this new source of resistance in tomato for protection against TSWV.

## Introduction

*Tomato spotted wilt tospovirus* (TSWV), a member of the genus *Tospovirus* in the family *Peribunyaviridae* and the order *Bunyavirales* (https://talk.ictvonline.org/taxonomy/p/taxonomy-history?taxnode_id=20162190), is one of the most important viruses that infects tomato (*Solanum lycopersicum*), worldwide^1^. The TSWV genome consists of three RNA segments designated as large (L), medium (M), and small (S)^2^. This virus has a broad host range, infecting ~1,090 plant species^3^. Under field conditions, TSWV spreads from plant to plant by multiple species of thrips, primarily the Western flower thrips (*Frankliniella occidentalis*)^4^. TSWV causes plant stunting and chlorotic or necrotic spots on leaves and fruits, resulting in yield losses that can exceed $1 billion annually in the U.S.^5^.

Host resistance is the most effective and economical means of managing any disease, including TSWV. Conventional tomato breeding often begins by screening germplasm resources, typically wild tomato relatives, to identify sources of resistance. Once identified, a resistant accession is backcrossed to cultivated tomato to introgress the resistance allele. The first resistance source to TSWV was found in *S. pimpinellifolium*^6^. Over the years, seven TSWV resistance loci have been identified, designated as the dominant and allelic *Sw-1a* and *Sw-1b*; three recessive genes: *sw-2*, *sw-3*, and *sw-4*; and three dominant genes: *Sw-5*, *Sw-6*, and *Sw-7*^7–10^. *Sw-5*, originally introgressed in the cultivar ‘Stevens’, is currently the primary source of TSWV resistance in commercial tomato varieties worldwide^11^. In addition to conferring a broad spectrum resistance to TSWV isolates, *Sw-5* also confers resistance to closely related tospoviruses, including *Tomato chlorotic spot tospovirus* (TCSV) and *Groundnut ringspot tospovirus* (GRSV)^12^. Unfortunately, several *Sw-5* resistance-breaking strains of TSWV have been identified in various regions around the world^13^, including the U.S. mainland^14^. Sequence comparison among TSWV isolates revealed that the ability of the virus to overcome *Sw-5* is associated with C to Y amino acid substitutions at position 118 (C118Y) and T to N substitutions at position 120 (T120N) in the TSWV movement protein (NSm). The NSm protein is responsible for cell-to-cell movement, tubule formation, symptomology, host-range determination and interactions with the TSWV N protein^14,15^. There is therefore an urgent need to utilize other TSWV resistance loci in place of, or along with, *Sw-5*. The *Sw-6* resistance locus confers only partial resistance under thrips inoculation and is effective against an even narrower range of TSWV isolates than *Sw-5*^16^. Alternatively, *Sw-7*, is reported to exhibit field resistance against various isolates of TSWV, including those that overcome *Sw-5*^17^. *Sw-7* was introgressed from *S. chilense* accession LA 1938 and is generally mapped onto chromosome 12^9,18^, but the molecular mechanism underlying this locus remains unknown.

In an effort to uncover the gene networks that are associated with *Sw-7* resistance, we performed comprehensive comparative analysis of global gene expression profiles in response to TSWV infection between a TSWV-susceptible parental line (Fla. 8059) and a *Sw-7* near isogenic line (with isogenicity estimated at 97.125% identity to the parental line Fla. 8059). From this analysis, 1,244 DEGs were identified between the two lines at five time points during disease progression from inoculation to symptom expression. Our findings provide a fundamental understanding of the virus-host interactions and identification of important candidate gene(s) for elucidation of the underlying mechanisms of *Sw-7* resistance against TSWV, which may have broad implications for characterization of the mechanism of resistance in other plant-virus systems.

## Results

### Summary of RNA-Seq datasets and differentially expressed genes between *Sw-7* and S-line

**Figure 1:**
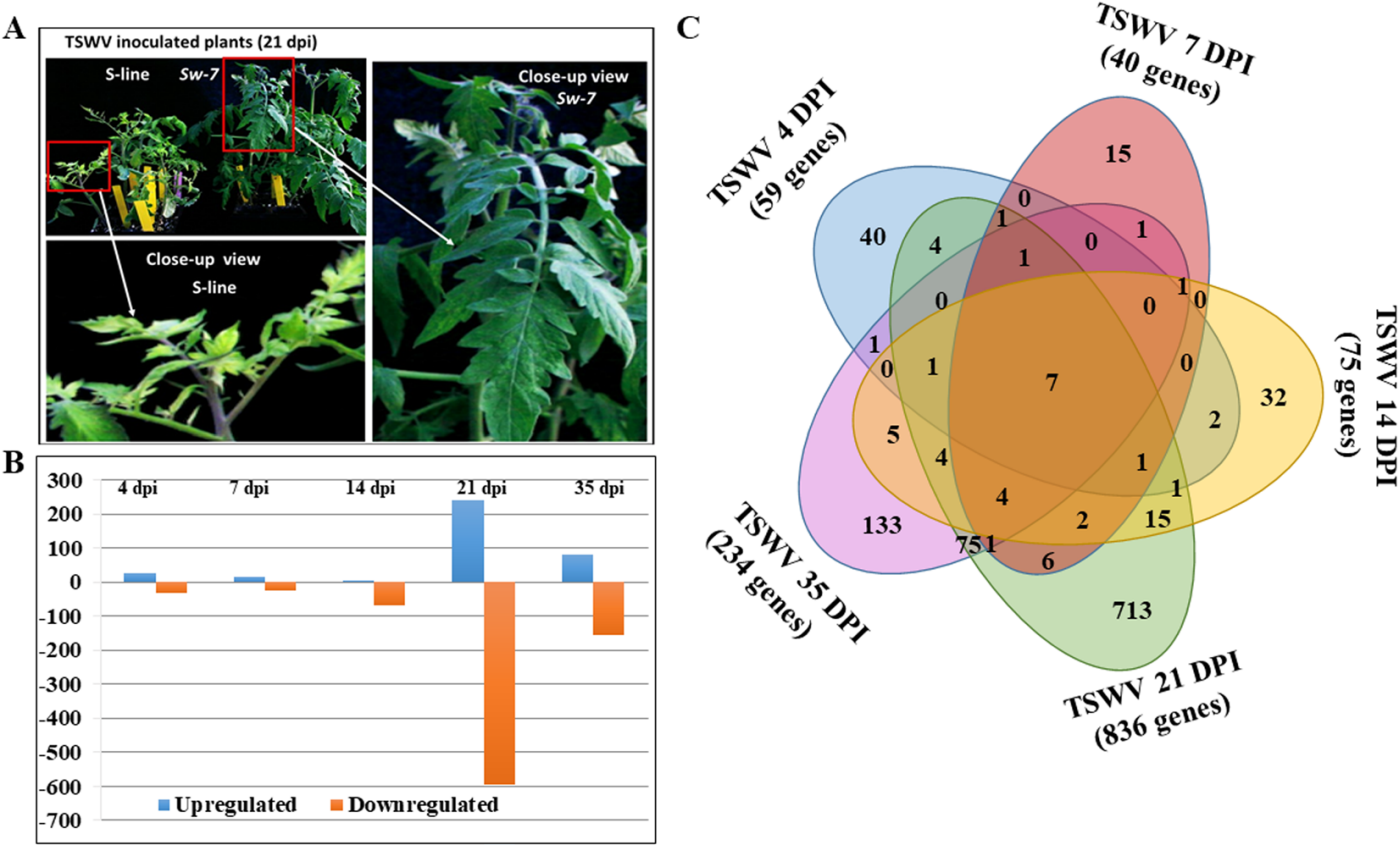
Differential gene expression between resistant *Sw-7* line and susceptible S-line after TSWV infection. (**A**) Plants of S-line (left), and the *Sw-7* at 21 days post inoculation (dpi) with TSWV (inset close view of a single leaf). (**B**) Numbers of differentially expressed genes in *Sw-7* line compared to S-line at 4, 7, 14, 21 and 35 dpi with TSWV. (**C**) Venn diagram showing the numbers of common, intersecting and specific DEGs at 4, 7, 14, 21 and 35 dpi with TSWV.

To provide a global view on differential gene expression between a near-isogenic line containing the *Sw-7* resistance locus (hereafter referred to as *Sw-7* line) and its susceptible recurrent parental line (Fla. 8059, hereafter referred to as S-line), comparative transcriptome profiling analysis was conducted using leaf samples collected throughout the virus infection process from inoculation to symptom expression. From these two lines, three biological replicate samples were taken at each of the five time points, 4, 7, 14, 21, and 35 days post inoculation (dpi). Typical disease symptoms, including chlorosis, mosaic, and necrotic lesions, were observed on the susceptible S-line plants at approximately 14–21 dpi. During the same period, symptoms were very mild to non-visible on TSWV-inoculated *Sw-7* line plants (Fig. 1A). Real-time RT-PCR confirmed the presence of TSWV in the inoculated leaves as early as 4 dpi in both *Sw-7* line (mean Ct: 27.02) and S-line plants (mean 27.43) (Supplementary Table S1), indicating virus infection had occurred and TSWV was replicating in the inoculated leaves. At 7 dpi, virus concentration continued to increase in the S-line (mean Ct: 22.46), but TSWV was nearly undetectable in systemic leaves in the *Sw-7* line (mean Ct: 35.01). At later time points of 14, 21, and 35 dpi, these trends continued, with high levels of virus accumulation in three S-line plants (mean Ct: 22.54, 16.88, and 22.48, respectively), and much lower virus concentrations in the *Sw-7* line plants (mean Ct: 33.04, 31.51, and 32.41, respectively) (Supplementary Table S1). These results indicated that although TSWV was initially capable of replicating in the inoculated leaves of the *Sw-7* plants, virus movement or replication was restricted and did not become systemic. Over time, disease expression in the *Sw-7* plants ranged from asymptomatic to mild disease symptoms with lower virus titer. On the other hand, the inoculated S-line plants exhibited severe disease symptoms with much higher virus titers in the systemic (upper uninoculated) leaves (Fig. 1 and Supplementary Table S1).

To achieve a comprehensive understanding on genes and pathways of tomato in response to TSWV infection, a total of 30 RNA-Seq libraries were constructed and sequenced. Differential gene expression was evaluated through an extensive comparative transcriptome analysis between the *Sw-7* line and the S-line plants. We used fastqc (http://www.bioinformatics.babraham.ac.uk/projects/fastqc/) to assess the quality of both raw and final cleaned RNA-Seq reads, and ensured that the cleaned reads were of high quality. Overall, an average of 15.3 million raw reads per library were obtained. After adapter trimming and removal of low quality reads and rRNA sequences, an average of 9.9 million high quality cleaned reads were obtained, with 85% of those reads mapped to the tomato genome (version SL2.5) (Supplementary Dataset S1). Values of Pearson’s correlation coefficients for all biological replicates were high, suggesting highly reproducible data generated by RNA-Seq (Supplementary Dataset S2).

Comparative analysis of gene expression levels revealed that out of the 34,727 genes predicted in the tomato genome, a total of 1,244 (3.58%) differentially expressed genes (DEGs) were identified between TSWV-infected *Sw-7* line and S-line plants during disease progression at 4, 7, 14, 21 and 35 dpi. Volcano plots were generated to illustrate distribution patterns of DEGs at each time point (Supplementary Fig. S1). Fewer genes were affected in the pre-symptom expression phase, as demonstrated at 4 and 7 dpi. Of 59 DEGs identified at 4 dpi (Supplementary Dataset S3), 27 were upregulated and 32 were down-regulated in the *Sw-7* line compared to the S-line. Similarly, of 40 DEGs identified at 7 dpi (Supplementary Dataset S3), 16 were upregulated and 24 were down-regulated (Fig. 1B). However, as days after inoculation elapsed, the number of DEGs increased, peaking at 21 dpi, the time when symptoms began to appear in the plants of the S-line (Fig. 1). Most of the DEGs affected by TSWV infection were not constant from one time point to another, and only seven DEGs intersected all 5-time points (Fig. 1C). Among these were a mannan-endo-1,4-beta-mannosidase and a chloroplastic group IIA intron splicing facilitator CRS1 which were both down-regulated, while the other five genes had unknown functions (Supplementary Table S2).

### Functional characterization of DEGs between the *Sw-7* line and the S-line in response to TSWV infection

**Figure 2:**
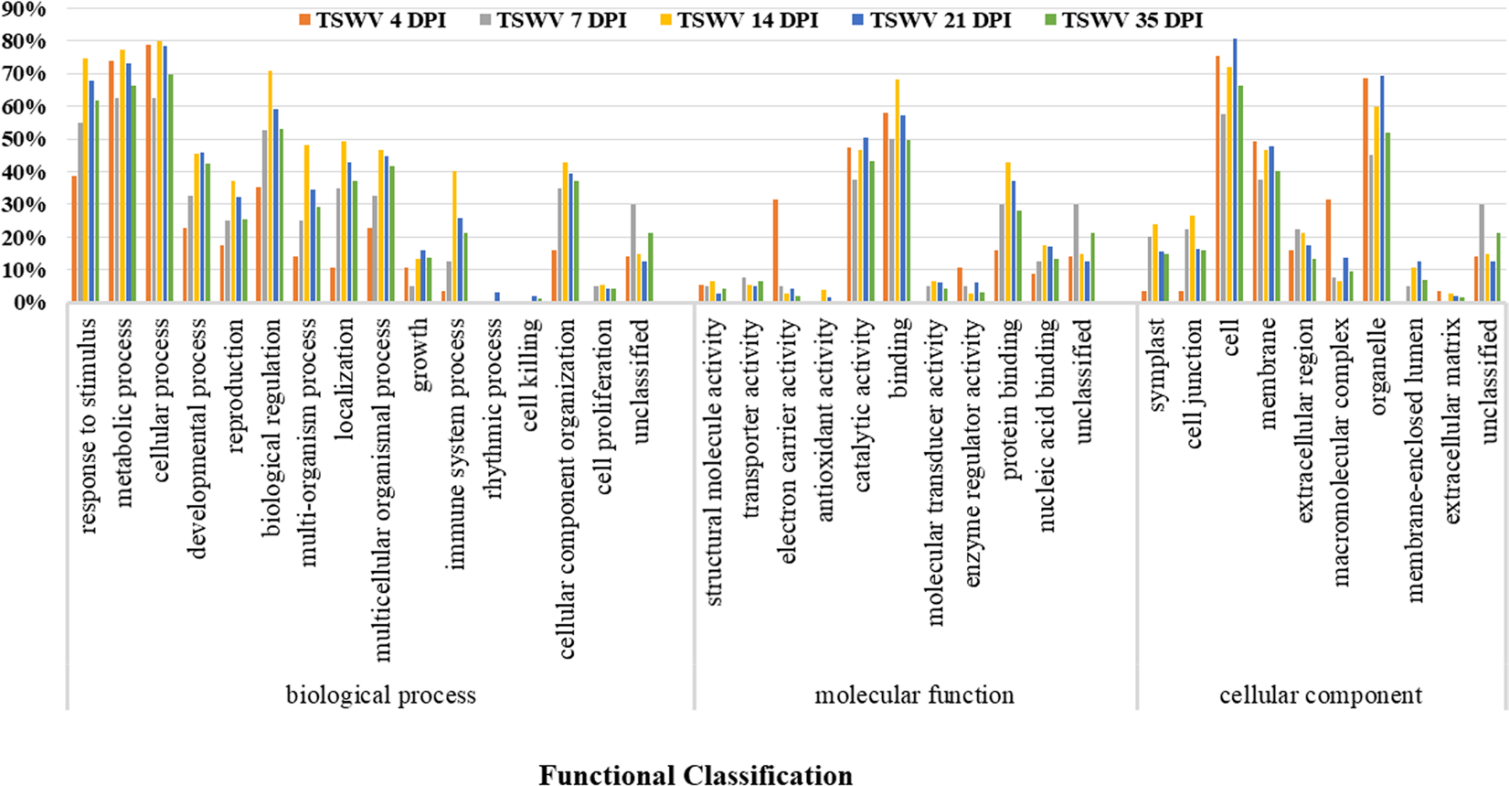
Gene Ontology (GO) functional classification of differentially expressed genes (DEGs). The percentage of genes assigned to each category were calculated at 4, 7, 14, 21 and 35 dpi, respectively.

DEGs were functionally classified using the broad gene ontology (GO) categories. In the biological process category, a large number of DEGs were related to response to stimulus, metabolic process, cellular process, and biological regulation (Fig. 2). In the molecular function category, the majority of DEGs were responsible for catalytic activity and binding. In the cellular component category, a high proportion of DEGs were related to cell, membrane and organelles (Fig. 2). GO enrichment analysis was also performed on DEGs at each time point (Supplementary Fig. S2). In genes upregulated in the *Sw-7* line at 4 dpi, GO terms including photosynthesis, electron carrier activity and chlorophyll binding were significantly enriched. However, in down-regulated genes at 4 dpi, only GO terms within the biological process category were enriched, including aldehyde and methylglyoxal metabolism. Although no enriched GO terms were identified from DEGs at 7 dpi, two genes encoding receptor-like proteins were upregulated in the *Sw-7* line. At 14 dpi, in five upregulated genes identified in the *Sw-7* line, no enriched GO terms were detected. At 21 dpi, in the stage of symptom expression, the majority of DEGs were induced in the S-line plants (Fig. 1). GO enrichment analysis revealed that these genes were related to plant defense and response to stimulus (Supplementary Fig. S2). A similar trend was observed in the post symptom stage at 35 dpi, when TSWV infection had resulted in severe symptom expression in the S-line plants. At this time point, genes in the photosynthesis, auxin homeostasis, and cellular carbohydrate metabolism pathways were upregulated in the *Sw-7* line plants, and the susceptible S-line plants showed enrichment of genes related to plant wound response. S-line plants were also enriched for genes involved in xyloglucan transferase activity; these genes play a role in the organization of cellulose-xyloglucan matrix, which control the strength and extensibility of the plant primary cell wall.

To gain a better understanding of the mechanism of resistance and of possible candidate genes that are involved in the *Sw-7* resistance response, several categories of genes, including those encoding nucleotide-binding site leucine-rich repeat (NBS-LRR) proteins, defense-related proteins, transcription factors, protein kinases, as well as those related to phytohormone signaling, cell wall, photosynthesis, gene silencing, and microRNA target genes, were further analyzed as follows.

### Host defense-related genes

GO term enrichment analyses of DEGs between *Sw-7* and S- line plants in response to TSWV infection revealed a total of 68 genes related to immunity, defense response, and disease resistance signaling molecules (Table 1). Among them, only two NBS-LRR genes were differentially expressed, and both were induced in the S-line plants at 21 dpi (Table 1).

**Table 1:** Selected differentially expressed defense-related, RNA silencing pathway and microRNA target genes in *Sw-7* compared to the S-line after TSWV inoculation.

*S. lycopersicum* accession	Annotation	4 dpi	7 dpi	14 dpi	21 dpi	35 dpi
**DEFENSE-RELATED GENES**						
**NBS-LRR Genes**						
Solyc08g006970	LRR, resistance protein	—	—	—	−2.396	—
Solyc01g014840	TIR-NBS-LRR, resistance protein	—	—	—	−3.474	—
**MLO-like Protein**						
Solyc03g095650	MLO-like protein	—	—	—	−3.322	—
**PATHOGENESIS-RELATED PROTEIN GENES**						
Solyc07g006710	Pathogenesis-related-1: PR-1	—	—	—	1.546	—
Solyc12g014310	PR-like protein	—	—	−2.322	—	—
**Defensin**						
Solyc07g007760	Defensin protein	—	—	—	−4.322	−2.184
Solyc07g006380	Defensin-like protein	—	—	—	−2.144	—
**Nodulin**						
Solyc11g012930	Nodulin family protein	—	2.336	—	—	—
Solyc05g005870	Nodulin MtN21 family	—	—	—	2.124	—
**Chitinase**						
Solyc07g005100	Chitinase-like protein	—	—	—	−3.474	—
Solyc05g050130	Acidic chitinase	—	—	—	−4.644	−2.737
Solyc02g082920	Endochitinase (Chitinase)	—	—	−3.184	−5.059	—
**Protease**						
Solyc10g086600	Subtilisin-like serine protease	—	—	—	—	4.563
**Peroxidase**						
Solyc06g050440	Peroxidase	—	—	—	−4.059	—
Solyc01g006300	Peroxidase	—	—	—	−2.120	—
**Osmotin-like protein, PR5**						
Solyc08g080670	Osmotin-like protein	—	—	—	3.065	—
**Protease Inhibitor**						
Solyc00g071180	Cysteine proteinase inhibitor	—	—	—	−6.644	—
Solyc03g097270	Cysteine proteinase inhibitor	—	—	—	−2.120	—
Solyc03g098740	Kunitz trypsin inhibitor	—	—	—	−3.837	—
Solyc03g098790	Kunitz-type	−1.889	—	—	−6.644	—
Solyc03g098780	Kunitz-type	—	—	—	−2.644	—
Solyc03g098760	Kunitz-type like protein	—	—	—	−5.644	—
Solyc09g089530	Proteinase inhibitor I	—	—	—	−4.322	—
Solyc09g089540	Proteinase inhibitor I	—	—	—	−5.648	—
Solyc09g089520	Proteinase inhibitor I	−4.644	—	—	−5.059	—
Solyc09g089510	Proteinase inhibitor I	—	—	—	−2.556	—
Solyc09g084470	Proteinase inhibitor I	—	—	—	−4.059	—
Solyc09g084480	Proteinase inhibitor I	—	—	—	−2.943	—
Solyc09g083440	Proteinase inhibitor I	—	—	—	−3.474	—
Solyc09g084490	Proteinase inhibitor I	—	—	—	−4.322	—
**OTHER DEFENSE GENES**						
**Glycine-rich protein (Inducer of PR-1)**						
Solyc06g061200	Glycine-rich protein TomR2	—	1.880	—	—	—
**Major latex-like protein**						
Solyc09g014530	Major latex-like protein	—	—	—	−4.322	—
Solyc08g023660	Major latex-like protein	—	−1.889	—	—	—
Solyc05g046150	Major latex-like protein	−2.737	—	—	—	—
Solyc04g005700	Major latex-like protein	−1.556	—	—	—	—
**Universal stress protein**						
Solyc09g011660	Universal stress protein	—	—	—	−2.396	—
Solyc04g014600	Universal stress protein	—	—	—	−3.059	—
Solyc01g057000	Universal stress protein	—	—	—	−2.943	−5.059
**TMV response**						
Solyc04g082960	TMV response-related	—	—	−3.059	−2.837	—
**Disease resistance**						
Solyc01g021600	Disease resistance response	—	—	—	−5.644	—
**Calmodulin**						
Solyc01g010020	Calmodulin	—	—	—	—	−3.184
Solyc02g091500	Calmodulin	—	—	—	−2.252	—
Solyc07g040710	Calmodulin-binding protein	—	—	—	—	—
Solyc03g113940	Calmodulin-binding protein	—	—	—	−5.644	—
Solyc03g119250	Calmodulin-binding protein	—	—	−2.474	—	—
Solyc02g088090	Calmodulin-like protein	—	—	−3.322	—	—
**Heat Shock Protein**						
Solyc06g076520	class I heat shock protein	—	—	—	−2.322	—
Solyc06g076570	class I heat shock protein	—	—	—	−3.644	—
Solyc06g076560	class I heat shock protein	—	—	—	−3.059	—
Solyc02g093600	class I heat shock protein	—	—	—	−5.644	—
Solyc04g014480	class I heat shock protein 3	—	—	—	—	−3.059
Solyc08g062450	class II heat shock protein	—	—	−3.837	—	—
Solyc03g113930	class IV heat shock protein	—	—	—	—	−3.644
Solyc11g020330	class IV heat shock protein	—	—	—	−4.644	−3.644
**F-Box Protein**						
Solyc**11g006740**	**F-box protein**	—	—	—	**−4.322**	**−3.644**
**GENE SILENCING PATHWAY GENES**						
Solyc02g069260	ARGONAUTE 1	—	—	—	−3.059	—
Solyc11g008540	SlDCL2b	—	—	—	−2.737	—
Solyc11g008530	SlDCL2d	—	—	—	−2.252	—
**MICRORNA-TARGET GENES**						
Solyc03g115850	miR164-NAC domain	—	—	—	−3.837	—
Solyc06g069710	miR164-NAC domain	—	—	—	−6.644	—
Solyc06g075510	miR172-AP2-like ERF	—	—	—	−2.252	—
Solyc10g006710	miR172-kinase receptor	—	—	—	−4.322	—
Solyc06g007320	miR396-Ubiquitin-activating enzyme E1	—	—	—	−2.120	—
Solyc01g005780	miR6022-LRR -RLP kinase	—	—	—	−5.644	—
Solyc01g006550	miR6022-LRR- RLP kinase	—	—	—	−4.644	—
Solyc01g009690	miR6022-LRR- RLP kinase	—	—	−3.837	−4.059	—
Solyc01g009700	miR6022-LRR- RLP kinase	—	—	—	−4.322	—
Solyc01g009930^a^	N/A -LRR- RLP kinase	—	—	—	−3.251	—

^a^N/A referred to be a non-annotated microRNA (It is not registered at the miRBase registry; it can be considered as a new microRNA).

Pathogenesis-related (PR) proteins often accumulate in plants upon pathogen attack and are typically induced as a defense response through systemic acquired resistance. Interestingly, a total of 27 PR protein family genes with differential expression between the *Sw-7* line and S-line plants were identified (Table 1). Among these, the pathogenesis related-1 (Solyc07g006710) gene was 1.5-fold higher expressed in the *Sw-7* line at 21 dpi, and two nodulin family genes were ~2-fold higher expressed in the *Sw-7* plants at 7 and 21 dpi. Two defensin (PR-12) genes were induced approximately 2- to 4-fold higher in the S-line at 21 dpi and 35 dpi. A total of 14 protease inhibitor genes, which belong to a pathogenesis-related protein subfamily (PR-6), were altered in our datasets. All of these were down-regulated (ranging from 2.1- to 6.6-fold) in the *Sw-7* line, with the majority (14) being induced in the S-line at 21 dpi. A subtilisin-like serine protease gene (Solyc10g086600) was upregulated 4.6 fold in the *Sw-7* line at 35 dpi. One gene encoding a member of glycine-rich protein (Solyc06g061200) was induced (~2-fold) in the *Sw*-*7* line at 7 dpi. Interestingly, a gene encoding an osmotin-like protein (OLP) was induced approximately 3-fold at 21 dpi in the *Sw-7* line (Table 1).

Three RNA silencing pathway genes, including one Argonaute 1 (*Ago1*) and two Dicer-like 2 (*DCL 2*), were down-regulated in the *Sw-7* line (Table 1). In addition, 10 microRNA target genes were identified, including miR164 (2 genes), miR172 (2 genes), miR396, miR6022 (4 genes), and an un-annotated or new miRNA. Most of these were induced in the S-line at 21 dpi, ranging from 2.1 to 6.6-fold in differential expression relative to that of the *Sw-7* line (Table 1).

### Transcription factors (TFs)

In this study, a large number of TF genes (78 genes) exhibited differential expression between the *Sw-7* line and the S-line plants (Table 2). Overall, only nine TF genes were up-regulated while 69 were down-regulated in the *Sw-7* line (Table 2). The nine induced TFs include three in the basic helix-loop-helix (bHLH) family, one in the bZIP family, one in the MADS-box family, two Myb transcription factors, one nuclear transcription factor Y subunit B-3, and one Cycloidea transcription factor. There were nine AP2-like ethylene-responsive transcription factors that were induced in the S-line (2–4 fold) at 21 dpi. Five bHLH transcription factors were altered, with three being induced in the *Sw-7* line at 21 or 35 dpi, and two being induced in the S-line. Interestingly, one of the bHLH genes was induced at a very high level (6.8-fold) in the *Sw-7* line (Table 2).

**Table 2:** List of differentially expressed transcription factors (TFs) in *Sw-7* compared to the S-line after TSWV inoculation.

*S. lycopersicum* accession	Annotation^a^	4 dpi	7 dpi	14 dpi	21 dpi	35 dpi
**AP2/ERF-AP2 Family**						
**Solyc**05g051380	AP2-like ethylene-responsive	—	—	—	−4.644	—
**Solyc**06g075510	AP2-like ethylene-responsive	—	—	—	−2.252	—
**Solyc**02g077370	Ethylene responsive TF-2	—	—	−5.059	—	—
**Solyc**03g093540	Ethylene responsive TF-1a	—	—	—	−2.396	—
**Solyc**03g093560	Ethylene responsive TF-2	—	—	—	−2.556	—
**Solyc**03g093610	Ethylene responsive TF-1b	—	—	—	—	−2.837
**Solyc**04g071770	Ethylene responsive TF-2a	—	—	—	−3.184	—
**Solyc**05g052410	Ethylene responsive TF-1	—	—	—	−5.059	—
**Solyc**11g012980	Ethylene responsive TF-9	—	—	—	−4.322	—
**BHLH and BZIP Family**						
**Solyc**02g087860	Transcription factor style2.1	—	—	—	1.840	—
**Solyc**03g121240	bHLH TF-like	—	—	—	—	2.384
**Solyc**04g007300	bHLH TF	—	—	—	6.759	—
**Solyc**07g005400	bHLH TF	—	—	—	−2.396	—
**Solyc**12g036470	bHLH TF	—	—	—	−4.644	—
**Solyc**01g109880	bZIP TF	—	—	—	−2.837	—
**Solyc**04g082890	bZIP TF	—	—	—	−1.889	—
**Solyc**07g062710	bZIP TF-family protein	—	—	—	1.807	—
**Solyc**10g078290	bZIP TF-family protein	—	—	—	−2.000	—
**Zinc Finger Protein Family**						
**Solyc05g009310**	ZF-CONSTANS-LIKE 16	—	—	—	−6.644	—
**Solyc**05g024010	ZF-CONSTANS-LIKE	—	—	—	−3.184	—
**Solyc**09g074560	ZF-CONSTANS-LIKE	—	—	—	−2.474	—
**Solyc**01g090840	Cys2/His2 ZF	—	—	—	−3.837	—
**Solyc**01g107170	Zinc finger protein	—	—	—	−3.059	—
**Solyc**06g075780	Cys2/His2 ZF	—	—	—	−4.059	−3.184
**Solyc**11g073060	ZF-family protein	—	—	—	−4.644	−5.059
**Solyc**06g071860	ZF-CCCH-67	—	—	—	−2.644	—
**Solyc**01g102980	Zinc finger-HD	−2.474	—	—	—	—
**Solyc**04g080490	Zinc finger-HD	—	—	—	2.395	—
**Solyc**09g005560	Zinc finger and SCAN	—	—	—	−6.644	—
**GARP, MYB, GRAS and Scarecrow Family**						
**Solyc**06g061030	GARP-ARR-B	—	—	—	−2.059	—
**Solyc**10g076460	Myb-like DNA-binding domain	—	—	—	−2.223	—
**Solyc**05g053090	GRAS family	—	—	—	−2.556	—
**Solyc**05g054170	Scarecrow-like 1	—	—	—	−2.644	—
**HB-HD-ZIP Family**						
**Solyc**02g063520	Homeobox leucine zipper	—	—	—	—	−2.252
**Solyc**03g082550	Homeobox leucine zipper	—	—	—	−3.322	—
**Solyc**05g051460	Homeobox-leucine zipper	—	—	—	−2.474	—
**Solyc**06g053220	Homeobox leucine zipper	—	—	—	−2.059	—
**Solyc**08g083130	Homeobox leucine zipper	—	—	—	−3.322	—
**Solyc**03g034150	Homeobox leucine zipper	—	—	—	—	−4.322
**HS-TF, LOB and MADS Family**						
**Solyc**02g090820	Heat stress TF-	—	—	—	−3.474	—
**Solyc**06g053960	Heat stress TF-A3	—	—	—	−2.474	—
**Solyc**03g119530	LOB domain protein 42	—	—	—	—	−2.943
**Solyc**04g077990	LOB domain protein 38	—	—	−2.396	—	−5.059
**Solyc**11g072470	LOB domain protein 1	—	—	—	−3.644	—
**Solyc03g114840**	MADS box	—	—	—	1.915	—
**MYB Family**						
**Solyc**03g093890	Myb-related TF	—	—	—	—	−2.737
**Solyc**05g048830	MYB TF	—	—	—	−2.737	-
**Solyc**06g053610	Myb-related TF	—	—	—	−2.644	-
**Solyc**07g008010	Myb TF	—	—	—	1.891	-
**Solyc**08g008480	Myb TF	—	—	—	2.118	-
**Solyc**06g005310	Myb TF	—	—	—	−2.737	-
**NAC Family**						
**Solyc**02g077610	NAC domain TF	—	—	—	−3.184	−3.322
**Solyc**02g093420	NAC domain TF	—	—	—	−2.837	−2.737
**Solyc**03g115850	NAC domain TF	—	—	—	−3.837	—
**Solyc**05g021090	NAC domain TF	—	—	—	−6.644	—
**Solyc**06g069710	NAC domain TF	—	—	—	−6.644	—
**Solyc**07g045030	NAC domain TF	—	—	—	−4.322	—
**Solyc**08g077110	NAC domain TF	—	—	—	−4.644	—
**Solyc**10g055760	NAC domain TF	—	—	−2.837	—	—
**Solyc**11g068620	NAC domain TF	—	—	−4.322	—	−2.644
**Solyc**12g013620	NAC domain TF	—	—	—	−4.644	−2.837
**WRKY Family**						
**Solyc**02g080890	WRKY TF-16	—	—	—	−3.474	—
**Solyc**04g051690	WRKY TF-16	—	—	−2.943	—	—
**Solyc**06g008610	WRKY TF-25	—	—	—	−2.120	—
**Solyc**07g051840	WRKY TF	—	—	—	−2.396	—
**Solyc**07g056280	WRKY TF-16	—	—	—	—	−2.737
**Solyc**08g062490	WRKY TF-16	—	—	—	−3.837	—
**Solyc**08g067340	WRKY TF	—	—	—	—	−3.474
**Solyc**08g067360	WRKY TF-9	—	—	—	−6.644	—
**Solyc**09g014990	WRKY-like TF	—	—	−2.252	—	—
**Solyc**09g066010	WRKY TF-25	—	—	—	−2.252	—
**Solyc**10g009550	WRKY TF	—	—	−3.184	−4.644	—
**Other Family members**						
**Solyc**05g056040	Auxin response factor 14	—	—	—	−2.184	-
**Solyc**07g007220	S/T phosphatase	—	—	—	−2.474	—
**Solyc**09g074760	Nuclear TF- Y subunit B-3	—	—	—	1.618	—
**Solyc**10g076180	Plant-specific domain	—	—	—	−5.059	—
**Solyc**12g014140	TF CYCLOIDEA	−3.644	—	3.474	—	—
**Solyc**09g005560	Zinc finger and SCAN	—	—	—	−6.644	—

^a^TF-Transcription Factor; S/T-Serine threonine; ZF-Zinc Finger.

On the other hand, four bZIP TFs were differentially expressed between *Sw-7* and S-line plants. In addition, eight zinc finger proteins (C2C2-CO, C2H2 and C3H family) were differentially expressed. All eight of these were induced in the S-line at 21 dpi, two of which were also induced at 35 dpi. Furthermore, six homeobox leucine zipper proteins (HB-HD-ZIP) were induced in the S-line, mostly at 21 dpi after symptoms had already appeared on the infected susceptible plants. Among the six differentially expressed MYB transcription factor genes, two were induced and four were suppressed in the *Sw-7* line. One nuclear transcription factor (NF-YB) and two Zinc finger-homeodomain protein 1 (zf-HD) genes were upregulated in the *Sw-7* line (Table 2).

### Protein kinases (PKs)

In the present study, only two of 42 protein kinase genes with differential expression were induced in the *Sw-7* line. These were a Pto-like serine/threonine kinase from the RLK-Pelle_CrRLK1L-1 family and a RLK receptor-like kinase in the family of RLK-Pelle_LRR-XII-1 (Table 3).

**Table 3:** List of differentially expressed protein kinases (PKs) in *Sw-7* compared to the S-line after TSWV inoculation.

*S. lycopersicum* accession	Annotation^a^	4 dpi	7 dpi	14 dpi	21 dpi	35 dpi
**CAMK_CAMKL, CAMK_CDPK Family**						
Solyc09g056430	Kinase family protein	—	—	—	−3.059	—
Solyc03g005330	CBL-interacting PK-20	—	—	—	−4.322	—
Solyc09g018280	Calcium/calmodulin-dependent	—	—	—	−2.474	—
Solyc03g113390	Calcium-dependent PK-1	—	—	—	−2.474	—
Solyc04g081910	Calcium-dependent PK	—	—	—	−2.120	—
**Pto, S/T, LRR and RLK family**						
Solyc01g109950	Pto-like, S/T PK, resistance protein	—	—	—	—	3.123
Solyc01g007990	RLK, Receptor like protein	—	—	—	−2.943	—
Solyc02g071800	Receptor like kinase, RLK	—	—	—	−2.000	—
Solyc02g080040	Receptor-like PK	—	—	—	−3.474	—
Solyc03g120110	S/T kinase receptor	—	—	—	−3.322	—
Solyc10g006710	S/T kinase receptor	—	—	—	−4.322	—
Solyc05g009040	Receptor-like PK	—	—	—	−4.059	—
Solyc07g006480	LRR receptor S/T PK	—	—	−3.184	—	—
Solyc03g123860	Receptor like kinase, RLK	—	—	—	−2.943	—
Solyc04g074000	Receptor like kinase, RLK	—	—	−3.322	—	—
Solyc04g074030	Receptor like kinase, RLK	—	—	−3.474	−3.059	—
Solyc11g017270	Receptor like kinase, RLK	—	—	−2.474	−2.737	—
Solyc03g111800	Receptor like kinase, RLK	—	—	—	−3.059	—
Solyc02g070890	Receptor like kinase, RLK	—	—	—	1.922	—
Solyc04g009640	Receptor like kinase, RLK	—	—	—	−3.474	—
Solyc04g014900	Receptor like kinase, RLK	—	—	—	−2.556	—
Solyc06g006020	Receptor like kinase, RLK	—	—	—	−4.322	—
Solyc06g048740	Receptor like kinase, RLK	—	—	—	−5.059	—
Solyc12g089020	Receptor-like protein kinase	—	—	—	−6.644	—
Solyc10g075040	Receptor-like protein kinase	—	—	—	−2.396	—
Solyc04g057930	Receptor-like kinase	—	—	—	−2.184	—
Solyc11g072660	Receptor PK-like protein	—	—	—	−2.396	—
Solyc01g028830	ATP binding; S/T kinase	—	—	—	−2.059	—
Solyc01g112220	Serine/threonine PK	—	—	—	−2.396	—
Solyc03g032150	Serine/threonine K-like protein	—	—	—	−2.059	—
Solyc04g011520	Serine/threonine K-like protein	—	—	—	−2.252	—
Solyc04g082500	ATP binding- S/T kinase	—	—	−4.059	−3.837	—
Solyc05g053930	ATP binding-S/T kinase	—	—	-	−2.252	—
Solyc03g078360	Receptor-like PK	—	—	—	−3.837	—
Solyc03g078370	Receptor-like PK	—	—	—	−2.943	—
Solyc07g055400	Receptor-like kinase	—	—	—	−3.322	—
Solyc05g008960	Receptor-like PK	—	—	−3.644	−4.322	—
Solyc05g009010	Serine/threonine PK	—	—	−2.252	−2.184	—
Solyc05g010530	Serine/threonine PK	—	—	—	−2.644	—
Solyc12g036330	Receptor-like PK	—	—	—	−3.474	—
**STE_STE11, TKL-Pl-4 Family**						
Solyc07g051870	Protein S/T K	—	—	—	—	−3.644
Solyc03g006400	Protein kinase	—	—	—	−3.644	—

^a^PK, protein kinase.

### Phytohormone signaling

In the present study, a total of 33 phytohormone-related genes were identified as differentially expressed (Table 4). Among them, one geranylgeranyl prophosphate synthase pathway gene was induced in both 21 dpi and 35 dpi time points. Several DEGs in the Gibberellin and IAA Pathways were either up-regulated or down-regulated. However, a group of auxin pathway genes were highly induced in the *Sw-7* line. In total, seven out of eight auxin pathway genes were induced in *Sw-7* ranging from 1.6 to 8.4 folds.

**Table 4:** Selected differentially expressed phytohormone-related genes in *Sw-7* compared to the S-line after TSWV inoculation.

*S. lycopersicum* Accession	Annotation^a^	4 dpi	7 dpi	14 dpi	21 dpi	35 dpi
**Cytokinin Pathway**						
Solyc04g016200	CK-O-glucoside biosynthesis	—	—	—	−2.556	—
Solyc10g079930	CK-O-glucoside biosynthesis	—	—	−2.837	—	—
**Ethylene biosynthesis Pathway**						
Solyc00g095860	ET biosynthesis	—	—	—	−3.322	—
Solyc01g095080	ET biosynthesis	—	—	—	−6.644	—
Solyc08g081550	ET biosynthesis	—	—	—	−3.184	—
Solyc09g010000	ET biosynthesis	—	—	—	−3.474	—
Solyc09g089580	ET biosynthesis	—	—	—	−2.474	—
**Geranylgeranyl Pyrophosphate Synthase Pathway**						
Solyc04g079960	geranylgeranyldiphosphate biosynthesis	—	—	—	1.541	1.727
**Gibberellin, IAA Pathways**						
Solyc03g006880	gibberellin biosynthesis	—	—	—	1.832	—
Solyc11g072310	gibberellin biosynthesis)	—	—	—	−1.888	—
Solyc11g011210	Gibberellin regulated protein	—	—	—	−4.059	—
Solyc06g007890	Gibberellin regulated protein	—	—	—	2.384	—
Solyc12g042520	Gibberellin-regulated family protein	—	—	—	−2.737	—
Solyc06g008870	GID1-like gibberellin receptor	—	—	—	−2.837	—
Solyc08g068480	IAA-amido synthetase GH3.8	—	—	—	1.787	—
Solyc01g107400	IAA-amido synthetase GH3.8	—	—	—	—	—
Solyc08g068490	IAA-amido synthetase GH3.8	—	—	—	1.761	−4.644
Solyc07g043590	IAA biosynthesis I	—	—	—	−1.889	—
Solyc06g073060	IAA biosynthesis II	—	—	—	−2.059	—
**Lipoxygenase Pathway**						
Solyc01g099160	jasmonic acid biosynthesis	—	—	—	−4.059	−2.644
Solyc01g099170	jasmonic acid biosynthesis	—	—	—	−4.059	−2.737
Solyc03g122340	jasmonic acid biosynthesis	—	—	—	−2.644	—
Solyc09g075860	jasmonic acid biosynthesis	—	—	—	−1.943	—
Solyc12g011040	jasmonic acid biosynthesis	—	—	—	−6.644	—
**Phenylpropanoid Pathway**						
Solyc02g079490	phenylpropanoid biosynthesis	—	—	—	—	1.687
Solyc02g093270	phenylpropanoid biosynthesis	—	—	−2.556	—	—
Solyc04g063210	phenylpropanoid biosynthesis	—	—	—	−3.644	—
Solyc06g074710	phenylpropanoid biosynthesis	—	—	—	—	1.727
Solyc09g082660	phenylpropanoid biosynthesis	—	—	—	—	−3.837
Solyc11g071470	phenylpropanoid biosynthesis	—	—	—	—	−4.644
Solyc11g071480	phenylpropanoid biosynthesis	—	—	—	—	−4.322
**Beta-carotenehydroxylase Pathway**						
Solyc03g007960	lutein biosynthesis	—	—	—	—	−2.737
Solyc03g007960	zeaxanthin biosynthesis	—	—	—	—	−2.737
**Other Auxin Pathway Genes**						
Solyc01g110770	Auxin-induced SAUR-like protein	—	—	—	1.795	—
Solyc11g011700	Auxin-induced SAUR-like protein	—	—	—	1.868	—
Solyc11g011680	Auxin-induced SAUR-like protein	—	—	—	2.101	—
Solyc04g052970	Auxin-induced SAUR-like protein	—	—	—	8.395	—
Solyc12g005310	Auxin-responsive GH3-like	—	—	—	1.604	—
Solyc03g082530	Auxin-responsive family protein	—	—	—	−6.644	—
Solyc11g011710	Auxin-responsive protein	—	—	—	2.928	—

^a^CK-Cytokinin, ET-stands for ethylene.

### Cell wall-related genes

A total of 18 cell wall modification genes were altered in our datasets (Table 5). Interestingly, the majority of these genes were down-regulated in the *Sw-7* line, which means they were highly induced in the S line. On the other hand, three in five pectinesterase genes were induced in the *Sw-7* line.

**Table 5:** List of differentially expressed cell wall pathway genes in *Sw-7* compared to the S-line after TSWV inoculation.

*S. lycopersicum* accession	Annotation	4 dpi	7 dpi	14 dpi	21 dpi	35 dpi
**Cellulose biosynthesis**						
Solyc04g016470	cellulose biosynthesis	—	—	—	−5.644	—
Solyc12g014430	cellulose biosynthesis	—	—	—	−4.644	—
**Cuticular wax biosynthesis**						
Solyc01g088430	cuticular wax biosynthesis	−1.599	—	—	—	—
Solyc07g053890	cuticular wax biosynthesis	—	—	—	−4.059	—
**Suberin biosynthesis**						
Solyc02g093270	suberin biosynthesis	—	—	−2.556	—	—
Solyc04g063210	suberin biosynthesis	—	—	—	−3.644	—
Solyc09g082660	suberin biosynthesis	—	—	—	—	−3.837
**Wax esters biosynthesis**						
Solyc07g053890	wax esters biosynthesis I	—	—	—	−4.059	—
Solyc09g005940	wax esters biosynthesis I	—	−1.786	—	—	—
**3-oxoacyl-cyl-carrier-p-synthase**						
Solyc03g122120	3-oxoacyl-cyl-carrier-p-synthase	—	—	—	−2.474	—
**Expansin**						
Solyc06g051800	Expansin	—	—	—	−6.644	—
**Pectate lyase**						
Solyc05g014000	Pectate lyase	—	—	—	2.057	—
Solyc02g093580	Pectate lyase	—	—	—	−5.644	—
**Pectinesterase**						
Solyc02g080210	Pectinesterase	—	—	—	1.674	—
Solyc02g080200	Pectinesterase	—	—	—	—	1.546
Solyc06g009190	Pectinesterase	—	—	—	−3.837	—
Solyc03g083770	Pectinesterase	—	—	—	−4.644	—
Solyc01g079180	Pectinesterase	—	—	—	1.669	—

### Functional characterization of the osmotin-like protein (OLP) PR-5

Based on the transcriptome analysis in the present study, several defense-related genes were induced in the *Sw-7* line, including the pathogenesis-related family osmotin-like protein (OLP, a PR-5 protein). To gain a better understanding of the role of this gene in the *Sw-7* resistance response, the OLP gene (PR-5) was chosen for evaluation through over-expression in the susceptible tomato cultivar ‘Moneymaker’. We initiated an *Agrobacterium* transformation with ~1,602 explants (leaf-disc) of ‘Moneymaker’, which resulted in producing ~50 plantlets in the selection media, from which we recovered 27 rooted plants. Among them, one T_0_ line was selected for multiplication by rooted cuttings and used for virus inoculation.

**Figure 3:**
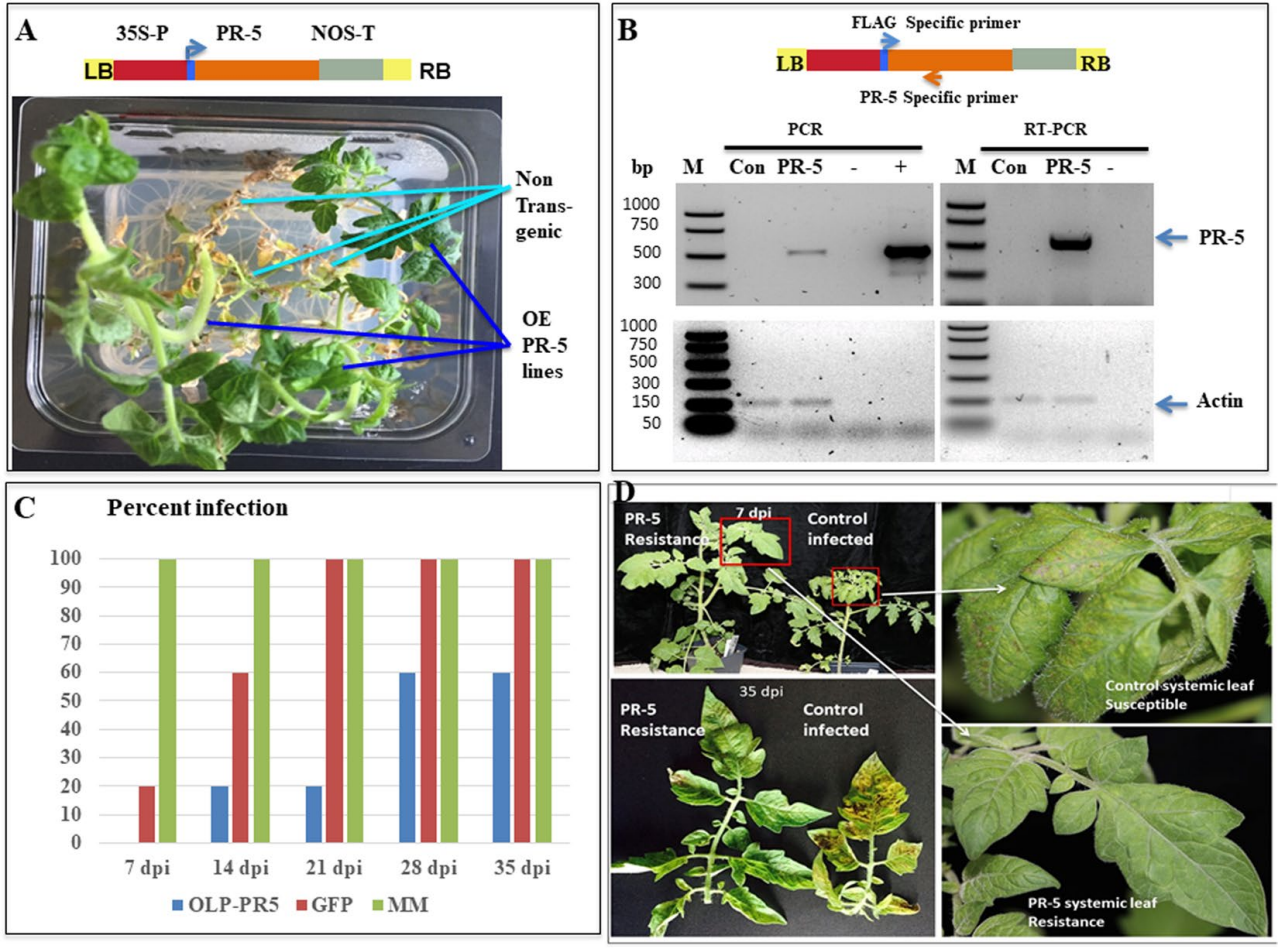
Functional validation of a candidate resistance gene PR-5 (OLP). (**A**) PR-5 gene was inserted between 35S and NOS terminator with an N-terminus FLAG tag. Transgenic plant lines regenerated on media containing phosphinothricin, but non-transformed plants could not survive (Top left). (**B**) PCR confirmation showed the presence of transgene (PR-5) and RT-PCR revealing the expression of transgene (PR-5) with positive (+), negative (−) controls and control (GFP) plants. Actin serves as the internal control in both cases for PCR reactions. (**C**) Percent of test plants infected as evaluated weekly over five weeks post inoculation on transgenic plants expressing OLP-PR5 gene, transgenic plants expressing GFP, and non-transformed ‘Moneymaker’ plants. (**D**) Evaluation of transgenic lines with resistance to TSWV: PR-5 over-expressing line (OLP-PR5) showed resistance to TSWV with no visible symptoms, whereas control plants (non-transformed Moneymaker) displayed chlorosis and necrotic spots upon TSWV inoculation.

Using T_0_ transgenic plants expressing OLP-PR5 and those with GFP as a negative control, we compared levels of resistance to mechanical inoculation of TSWV in a greenhouse (Fig. 3). The typical disease symptoms of TSWV infection were observed as early as 7–14 dpi on the non-transformed ‘Moneymaker’ (MM) plants or transgenic plants with GFP, but transgenic OLP-PR5 plants showed no visible symptoms (Fig. 3). At 7 dpi, real-time RT-PCR detected the presence of TSWV on 100% of the control MM plants and 20% of GFP plants. In the case of OLP-PR5 plants, virus infection was delayed for at least one week and detected in only 20% of test plants at 14 dpi. At 21 dpi, 100% of control plants were infected, but only 20% of OLP-PR5 plants tested positive for TSWV. Likewise, when the bioassay concluded at 35 dpi, still only 60% of OLP-PR5 test plants were infected (Fig. 3).

## Discussion

Using a near isogenic line generated through five backcrosses to the recurrent parental line, Fla. 8059, we developed a TSWV-resistant *Sw-7* line, which has a highly similar genetic background (97.125%) to the susceptible parental line (Hutton *et al*., unpublished). Therefore, differential gene expression profiles identified between *Sw-7* and S-line would be most likely to have resulted from their differential responses of the *Sw-7* locus to the TSWV infection. Although TSWV is a thrips-transmitted virus, it can also infect tomato via mechanical inoculation^19^. In the current study, we have followed the standard mechanical inoculation protocol established for TSWV in tomato. Our experiments revealed the effectiveness of the mechanical inoculation, since parallel inoculations on both S-line and *Sw-7* line plants showed similar virus load on the inoculated leaves at 4 dpi, but this load increased over time in the S-line plants relative to *Sw-7* plants (Supplementary Table S1).

By analyzing differential gene expression profiles between the *Sw-7* line and the S-line, we achieved a broad dynamic view of global gene expression and identified a number of induced defense-related genes in the *Sw-7* line. Interestingly, relatively fewer genes exhibited differential expression during the early virus infection stages (59 and 40 DEGs at 4 and 7 dpi, respectively). Many more genes with differential expression were observed at 21 dpi (836 DEGs) and 35 dpi (234 DEGs), at which time points visible disease symptoms had appeared on the susceptible S-line plants, but little to no symptoms were observed on the *Sw-7* line plants. Therefore, it is not surprising that the majority of DEGs at 21 dpi and 35 dpi were down-regulated in the *Sw-7* line. It is likely that at the peak of a virus infection, greater engagement of virus and host plant interactions had occurred in the susceptible S-line plants. For the resistant *Sw-7* plants, a greater proportion of genes with stronger expression at later time points were related to photosynthesis (13/14 genes) (Supplementary Table S3). In contrast, the inoculated susceptible S-line plants experienced significant changes in gene expression related to a variety of general immune system and defense response pathways, including phytohormone synthesis. Therefore, based on the various gene expression patterns, we were able to group and classify certain genes or gene families which were induced in the *Sw-7* line and associated with positive regulation of virus resistance. On the other hand, another group of genes or gene families were induced in the S-line and were likely involved in symptom induction as well as in defending plants for survival from the virus infection. Finally, a third group of genes and gene families had split roles, some members were induced in the *Sw-7* line and others in the S-line. Therefore, these genes might play dual roles in both positive and negative influence on virus resistance (Supplementary Fig. S3).

Transcription factors are proteins that control the rate of transcription, once bound to a specific DNA sequence. In the current study, some transcription factors were up-regulated, whereas many others were under down-regulated in the *Sw-7* line. These results were in a general agreement with the transcriptome profiling of bean common mosaic virus (BCMV) infection in common bean^20^. The expression of certain genes could be dynamic or zig-zag, as shown here in the expression of the TF Cycloidea which is profiled as down-regulated at 4 dpi and upregulated at 14 dpi. At this stage, we are unsure of its function in responding to the virus infection, which would need to be further characterized. Zinc finger proteins have been shown to play a key role in disease resistance, particularly in virus resistance^21^. Interestingly, a large number of zinc finger proteins were induced in the S-line, at the time when plants began to show disease symptoms. This indicates a likely stronger antiviral response in the infected plants, which could have been defeated by viral-pathogenicity factors leading to enhanced symptoms.

Phytohormones are signal molecules produced within the plant cells that regulate plant growth and development^22^. Plant hormones can vary as a response to pathogen infection. During virus infection, many plant hormone-signaling molecules are either suppressed or induced, which, in turn, affect normal plant growth, resulting in disease-like appearance, such as developmental defects and/or plant stunting^23^. Auxins have key roles in determining patterns of plant development and growth, which has four families: glutathione S-transferases, auxin homeostasis proteins like GH3, SAUR genes, and Aux/IAA. In *Arabidopsis*, auxin (Aux/IAA) mutant constitutively represses auxin response that leads to suppression of plant growth^24^. Upon TSWV infection, the S-line plants showed a reduction in plant growth, which corresponded with the reduction in auxin pathway gene expression. In studying the mechanism of resistance to TYLCV, Yang and colleagues^25^ identified a MADS-box transcription factor as one of the candidate genes for *Ty-2* resistance in tomato. Interestingly, in the present study, MADS-box genes were induced in the *Sw-7* resistance to TSWV (Supplementary Fig. S3), implicating a possible involvement of a MADS-box gene in *Sw-7* resistance.

Protein kinases (PKs) play a major role in disease resistance through phosphorylation of the interacting proteins to trigger active or functional processes. Although there are only a small number of PKs induced in the *Sw-7* plants, previous studies^26,27^ have shown that a Pto-like serine/threonine kinase protein^26^ was induced in tomato with resistance to a bacterial disease. A large number of PK genes induced in the S-line during the virus infection may play a function by activating currently unknown susceptible host factors or downstream signaling components to promote symptom expression. A higher number of PK genes induced in the S-line plants pointed to a likely stronger antiviral response in the infected plants as they attempt to fight back for survival from the virus infection.

Our analysis revealed the induction of 10 microRNAs in the S-line. Non-coding RNAs (ncRNAs), including microRNAs and long ncRNA, play key roles in regulating gene expression in plants and animals^28^. Such induction may result from a physical binding (sequester) of the TSWV-encoded suppressor protein (NSs) to microRNAs, leading to an elevated expression of the target genes and as a consequence enhanced virus accumulation in the S-line.

NBS-LRRs are a major category of disease resistance genes (R-genes) in plants, which have been classified into two sub-families: TIR-NBS-LRR and CC-NBS-LRR^29^. Typically, there are hundreds of diverse NBS-LRR genes in a plant genome^30^. However, in the current study, only two NBS-LRR genes showed differential expression, both down-regulated in the *Sw-7* line at 21 dpi, suggesting NBS-LRR genes are not likely the candidate resistance gene for *Sw-7* against TSWV infection.

Induction of PR genes leads to local and systemic defense gene activation, which could restrict virus movement. Previous study revealed PR proteins could be involved in resistance against fungi, bacteria and viruses^31^. A recent report demonstrated that TSWV-derived siRNAs can effectively silence the host transcripts, such as ERF, NBS-LRR class R-genes and heat stress transcription factors^32^. In addition, viroid infection in tomato revealed viroid triggered immune responses, in particular, induction of host calcium-dependent protein kinases (CDPKs) PR1 and WRKY^33^. Defensins are small cysteine-rich basic proteins found in animals and plants that function as host defense peptides against pathogens (including fungi, bacteria and viruses) and are considered part of the innate immune response^34^. Nodulins are also among the PR protein family of genes and are important for transport of nutrients, amino acids and hormones for plant development, as well as for pathogen fitness in host colonization^35^. Nodulins are also considered to be resistance marker proteins induced by plants in response to pathogenic bacterial infection^36^. In the current study, *PR-1* was induced in the *Sw-7* line, which might play an important role in resistance against TSWV infection. This discovery is important, as *PR-1* is a marker gene for disease resistance and it utilizes callose induction and deposition in the cell wall to restrict virus cell-to-cell movement^37^. Tomato and tobacco PR-1 proteins are also shown to have an antifungal activity against *Phytophthora infestans*^38^. The glycine/proline rich proteins have crucial roles in pathogen resistance by inducing PR proteins. Previous studies have demonstrated that glycine-rich proteins play a role in lignin biosynthesis and/or deposition^39^, and more importantly in enhancing callose deposition in the cell wall to block virus spread^40^. It is possible that the mechanism of resistance by *Sw-7* is through blocking or slowing down virus cell-to-cell or systemic movement. This hypothesis is also supported by our quantitative measurement of virus titers in the inoculated leaves of both *Sw-7* and S-line plants, which showed similar levels of virus titer in early infection stage at 4 dpi, but a gradual reduction in titer in systemic leaves of the *Sw-7* line plants relative to S-line plants (Supplementary Table S1).

**Figure 4:**
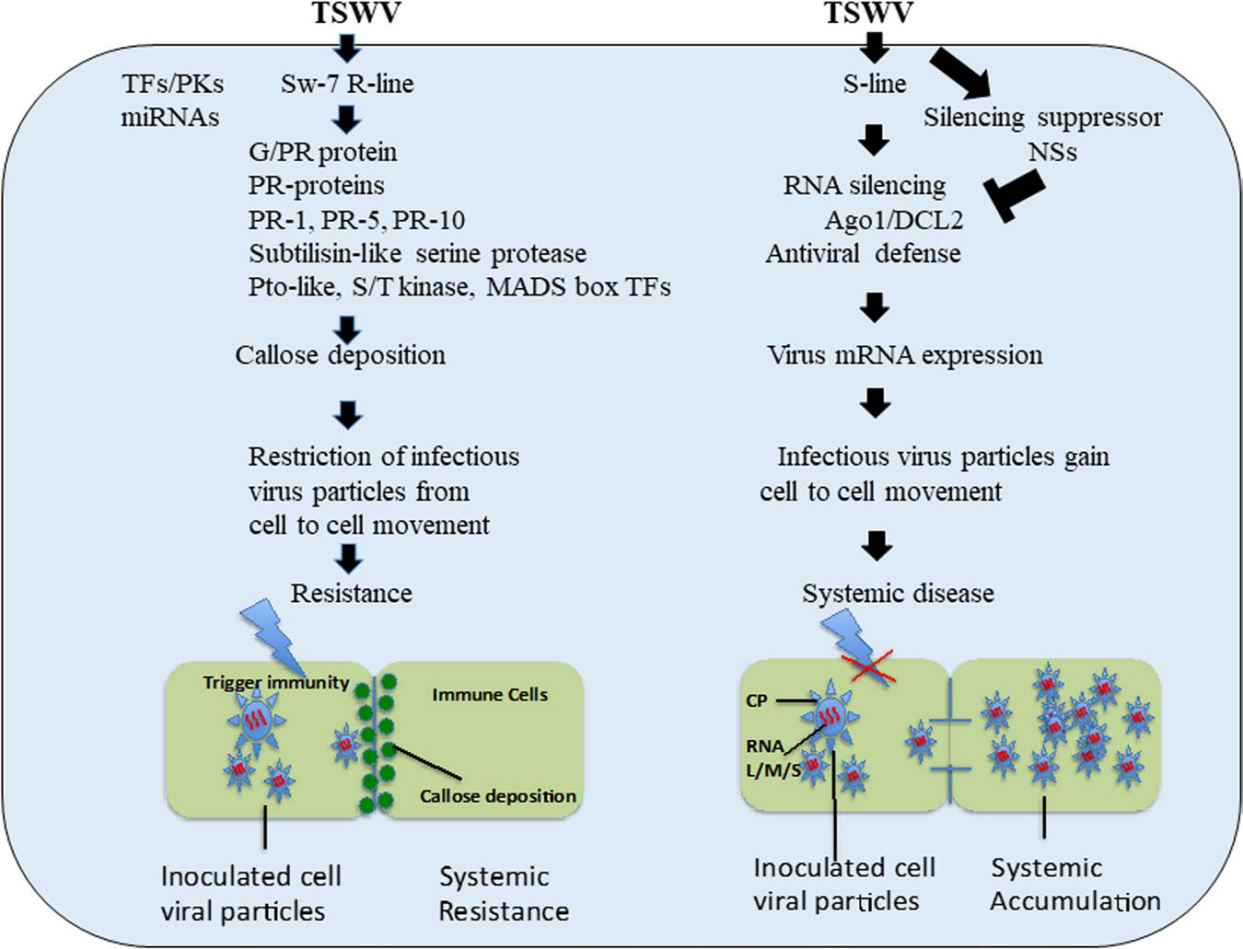
A schematic model illustrating the predicted mechanisms of virus resistance to TSWV in *Sw-7* tomato plants or of symptom expression in the susceptible (S) plants. The *Sw-7* resistance requires defense-related signaling molecules, including pathogenesis-related 1 (PR-1) protein, pathogenesis-related 5 (PR-5) (osmotic-like protein), glycine/proline rich protein (GRP), nodulin (PR-10), Pto-like R-gene (bacterial resistance), MADS box transcription factors (candidate *Ty-2* gene), and subtilisin serine protease, all of which showed elevated expression in the *Sw-7* line relative to S-line. The potential functional roles of the above stated genes, and signaling pathways including GRP-triggered PR proteins, are to actively communicate to neighboring cells, resulting in callose, lignin, and suberin deposition to the cell wall and leading to restricted cell-to-cell movement of TSWV. This in turn leads to the resistance phenotype in the *Sw-7* plants. For the susceptible response in the S-line, we speculate that the virus-encoded molecular factors would suppress the host immune pathways, leading to TSWV replication, transcription and translation. Abundance of viral RNA accumulation in the cells would trigger the expression of RNA silencing pathway genes in the S-line, including Argonaute 1 (Ago1) and Dicer-like 2 (DCL 2), resulting in antiviral defense. In the meantime, TSWV-encoded silencing suppressor protein (NSs) would suppress (sequester) the host antiviral defense pathway, leading to over-accumulation of viral particles. This in turn results in the opening of the cell wall/plasmodesmata to virus cell-to-cell and systemic movement, producing the disease phenotype.

Interestingly, another member of PR proteins, OLP from PR-5, was also induced. Induction of an OLP in plants in response to a viroid pathogen^41^ has been demonstrated, and PR-5 also plays a defense role against the fungus *P. infestans*^42^. Previous studies have also demonstrated that transgenic plants overexpressing OLP are tolerant to other stress factors such as salt, drought, cold, and bacterial and fungal pathogens^42–48^. Although PR-5 is involved in multiple bacterial/fungal resistance, its involvement in virus resistance has not been characterized. Given the high induction in gene expression at 21 dpi when the susceptible plants were at the peak of showing disease symptoms, we suspected that it may also be involved in *Sw-7* resistance to TSWV. To investigate its functional role in the defense response of *Sw-7* against TSWV, we overexpressed OLP into a susceptible tomato line. Interestingly, resistance evaluation demonstrated that the over-expression transgenic plants showed moderate resistance to TSWV infection in comparison to the control plants. We propose a *Sw-7* resistance model which involves OLP-PR5 to restrict virus movement from cell to cell through induction of callose deposition in the cell wall, resulting in virus resistance to TSWV (Fig. 4). Although it is quite clear that PR-5 was involved in the *Sw-7* resistance to TSWV, it is not likely the actual resistance gene since over-expression transgenic plants did not offer the same level of resistance as its natural parent. Therefore, the *Sw-7* gene (genes) remained to be identified. The discovery in association of a PR-5 gene for *Sw-7* resistance against TSWV would offer an opportunity in future studies to determine whether PR-5 and *Sw-7* have a direct or indirect interaction. Our future studies will also involve characterization of other identified PR-related candidate genes as well.

## Materials and Methods

### Plant materials and generation of a near-isogenic line of *Sw-7* with resistance to TSWV

Plant materials used for transcriptome experiments included the TSWV-susceptible inbred line, Fla. 8059^49^, and a *Sw-7* near-isogenic line (*Sw-7* NIL) in the Fla. 8059 background. The NIL was developed by crossing Fla. 8059 with the *Sw-7* donor line, Fla. 8516, followed by five backcrosses to the recurrent parent, Fla. 8059. Selection for the *Sw-7* introgression was accomplished using a linked SCAR marker designed from the CT99 RFLP^50^. CT99 is located within the interval on chromosome 12 to which the *Sw-7* locus is mapped^51^, and this location has been confirmed by recent fine-mapping (Hutton, unpublished data). Primer sequences (5′ to 3′) for the SCAR marker are F: GAAGGTGCCGACGGTGTA, R: AGGAATCAAGGTAAACCACCA. Amplicon sizes are 285 bp for the *Solanum chilense* allele and 241 bp for the *S. lycopersicum* allele. Seeds of the *Sw-7* NIL were produced by self-pollinating plants from the BC_5_F_1_, and then saved from BC_5_F_2_ plants that were homozygous for the *Sw-7* introgression.

### TSWV culture collection and maintenance

TSWV culture was collected from local infected tomato plants in Charleston, SC, and maintained on tomato ‘Moneymaker’ plants in an insect-proof bug-dorm in an environment-controlled greenhouse maintained at 26 °C with 14 h daylight and 10 h dark. Systemic infected leaf tissues were collected and tested for the presence of TSWV using enzyme-linked immunosorbent assay (ELISA) following the manufacturer’s instructions (Agdia, Elkhart, IN).

### Mechanical inoculation of TSWV on tomato

Seeds of the *Sw-7* NIL and its recurrent parent Fla. 8059 were germinated in separate plastic pots (10 cm) filled with Profession Growing Mix (Sungro, Agawam, MA) in an environment-controlled greenhouse. Mechanical inoculation was conducted on tomato seedlings at the 2–3 leaf stage^19^. Virus inoculum was prepared by grinding a small piece of TSWV-infected leaf tissue in a plastic bag with addition of tissue extraction buffer [0.01 M sodium phosphate buffer (pH 7.0) with 0.4% β-mercaptoethanol] in a final dilution 1:10 (w/v). Mechanical inoculation was performed by using a cotton swab (Q-tips) soaked with the inoculum and gently rubbed on the surface of tomato leaves that had been dusted with Carborundum powder (600 mesh). The inoculated plants were maintained in a greenhouse to monitor for symptom expression.

### Sample collection and real time quantification of TSWV

Virus-inoculated plants were maintained and monitored for symptom expression up to 35 dpi. TSWV-inoculated leaf tissues were collected at 4, 7, 14, 21 and 35 dpi, respectively. For sample collection, a small piece of leaf tissue (500 mg) from inoculated leaf (*Sw-7* and S-line) was collected at 4 dpi and subsequent collections were performed on systemic young leaf at other time points (7, 14, 21 and 35 dpi). Inoculated plants were tested to confirm for the presence and concentration of TSWV using real-time RT-qPCR^52^. RT-qPCR was performed using the TaqMan probe 5′HEX-AAATCTAAGATTGCTTCCCACCCTTTGATTCAA-BHQ, with forward primer 5′GCTTGTTGAGGAAACTGGGAATT and reverse primer 5′AGCCTCACAGACTTTGCATCATC^52^ located in the N gene of TSWV and Takara One Step PrimeScript RT-PCR Kit (Clontech, USA) following manufacturer’s instructions. The One-step RT-qPCR reaction was carried out on a Stratagene MX3000P Real-Time PCR machine (Agilent, USA), under the following conditions: 50 °C for 30 min, denaturation at 95 °C for 2 min, followed by 40 cycles of 95 °C for 1 min and 55 °C for 30 sec.

### Generation of plant transformation constructs

To functionally characterize some defense-related genes that are potentially contributing to TSWV resistance in the *Sw-7* line, tomato OLP (PR5) gene was selected for evaluation. A synthetic gene (OLP or GFP) was designed (IDT, Coralville, IA) and inserted into pENTR-D TOPO vector and transformed into Top 10 Chemically competent cells (Invitrogen, USA). Plasmid DNA with inserts from selected colonies were confirmed through Sanger sequencing. Construct was recombined with Gateway vector PEG101 using clonase (Invitrogen, USA) between the Cauliflower mosaic virus (CaMV) 35S promoter and nopaline synthase (NOS) terminator. The sequence confirmed OLP and GFP inserted binary vectors were mobilized into *Agrobactrium tumefaciens* strain LBA4404 by electroporation and selected on YM agar containing kanamycin for PEG101 selection and Streptomycin for *Agrobacterium*.

### *Agrobacterium*-mediated transformation and confirmation of transgenic plants

We followed an efficient protocol of tomato transformation and selection of transgenic plants^53^ with some modifications. Briefly, seeds of tomato “Moneymaker” after sterilization were germinated on a MS medium agar plate. Leaves and cotyledons of 2- to 3-week-old ‘Moneymaker’ seedlings were cut under sterile conditions to make small explants of about 2–3 mm. These explants were incubated for 10–15 min with the *Agrobacterium* suspension culture (infection solution). After incubation, explants were quickly wiped on a sterile filter paper and then transferred to a co-cultivation medium. After two days (48 h) of co-cultivation in the dark at 24 C, the explants were transferred to Petri dishes containing microshoot induction (MI) medium. After 3 weeks, the callus-forming explants that produced microshoots were cut and transferred to the shoot elongation medium. Shoots of 1/1.5 cm long after approximately three weeks of growth were cut and transferred into the rooting medium. Rooted plantlets were transferred into soil and maintained at 25 °C in a growth chamber. Confirmation of transgenic events in the regenerated plants was tested after 3–4 weeks of growth. The transgenic plants were self-pollinated. The T1 seeds were germinated on a MS basal medium containing 1 mg/L Phosphinotricin for selection. Surviving germinated seedlings were transferred to pots containing sterile soil and maintained in a glasshouse at 28–29 °C and 80–90% relative humidity. Transgene was confirmed by gene specific PCR and gene expression confirmed by RT-PCR using FLAG specific 5′- forward primers (KL17-151 FLAG-F:GACTACAAAGACGATGACGACA) and OLP specific reverse primers (KL14-397 OLP-1R:GCAACACATTGAATTGGATGACATT). For the internal control, an actin primer pair (forward primer KL17-071 03g078400F: TTGCTGGTCGTGACCTTACT and reverse primer KL17-072 03g078400R: TGCTCCTAGCGGTTTCAAGT) were used.

### Evaluation of OLP-PR5 transgenic tomato plants for resistance to TSWV

To evaluate transgenic plants over-expressing the OLP-PR5 gene for their resistance against TSWV, five rooted plants (in 4–5 leaf stage) regenerated from PCR-confirmed OLP-PR5 transgenic T_0_ lines, along with similarly developed transgenic plants expressing GFP or non-transgenic ‘Moneymaker’ plants were mechanically inoculated with TSWV using the same method as described above. In addition to observe symptom expression on the inoculated plants, virus titers accumulated on systemic leaves in each of the test plants were also measured quantitatively using real-time RT-qPCR as described above^52^ using leaf tissue samples collected on 7, 14, 21, 28 and 35 dpi (Supplementary Table S4).

### RNA extraction

Total RNA was extracted using TRIzol reagents (ThermoFisher Scientific, USA) from 500 mg of freshly collected leaf tissue in aplastic sample extraction bag and homogenized using Homex-6 homogenizer (BioReba, Swizerland) following manufacturer’s instructions. The DNase I-treated RNA was resuspended in NANOpure® water and its concentration measured with a NanoDrop spectrophotometer (ThermoFisher Scientific, USA). The cleaned DNA-free high quality RNA was also confirmed in a 1X bleach gel^54^.

### RNA-Seq library preparation, Illumina sequencing and data analysis

Strand-specific RNA-Seq libraries were constructed using the protocol described^55^ and sequenced on an Illumina HiSeq 2500 system using the single-end 100-bp mode. Raw RNA-Seq reads were processed to remove adaptor and low quality sequences using Trimmomatic^56^. RNA-Seq reads were then aligned to the ribosomal RNA database^57^ using Bowtie^58^ and the mapped reads were discarded. The remaining high-quality cleaned reads were aligned to the tomato Heinz genome (The Tomato Genome Consortium, 2012) using HISAT^59^. Following alignments, raw counts for each tomato gene were derived and normalized to reads per kilobase of exon model per million mapped reads (RPKM). Raw counts were fed to the DESeq package^60^ to identify genes differentially expressed between the *Sw-7* line and the S-line at each time point. Genes with adjusted p values less than 0.05 and fold changes greater than or equal to 1.5 were identified as DEGs.

The identified DEGs were uploaded into the Plant MetGenMAP system^61^ to identify enriched gene ontology terms, functional classifications, and biochemical pathways. Overlapping analysis of DEGs was performed with an online tool (http://bioinformatics.psb.ugent.be/webtools/Venn/). Summary plots of GO enrichment were created using Revigo^62^. The Tomato Functional Genomics Database^63^ and the iTAK database^64^ were used to identify plant transcription factors, receptor-like kinases, and miRNA targets. Identification of other genes of interest was performed using standalone BLAST^65^ by comparing homologs to genes of interest from *Arabidopsis* and *S. lycopersicum* in conjunction with utilizing annotated GO terms of tomato genes^66^ and manual annotation.

## Supplementary information

Supplementary Information

Supplementary Dataset S1

Supplementary Dataset S2

Supplementary Dataset S3

## Data Availability

RNA-Seq datasets were submitted to SRA database with the accession No. SRP119544.
