# Supplementary material for: Comprehensive transcriptome analysis and functional characterization of PR-5 for its involvement in tomato Sw-7 resistance to tomato spotted wilt tospovirus
Source: Sci Rep. 2019 May 21;9:7673. doi: 10.1038/s41598-019-44100-x (PMC6529424; doi:10.1038/s41598-019-44100-x)
Supplement: Supplementary file 1 — Supplementary Information [file 41598_2019_44100_MOESM1_ESM.pdf]

Title: Comprehensive transcriptome analysis and functional characterization of PR-5 for its involvement in tomato *Sw-7* resistance to tomato spotted wilt tospovirus

Supplementary Information

Author list: Chellappan Padmanabhan[1], Qiyue Ma[2], Reza Shekasteband[3], Kevin. S. Stewart[1], Samuel F. Hutton[3], John W. Scott[3], Zhangjun Fei[2,4*] and Kai-Shu Ling[1*]

SUPPLEMENTARY TABLES:

Supplementary Table S1. Evaluation of virus accumulation on each test plant of S-line and Sw-7 line on days post inoculation using TSWV-specific real-time quantitative RT-PCR

Treatment	Test plants and replicates	Ct (dRn)[d]	Mean Ct _replicate	Mean Ct _treatment
S-1 control [a]	before inoculation 1[a]	No Ct	No Ct	No Ct
	before inoculation 1	No Ct		
	before inoculation 2	No Ct	No Ct	
	before inoculation 2	No Ct		
	before inoculation 3	No Ct	No Ct	
	before inoculation 3	No Ct		
Sw-7 [b]	before inoculation 1[b]	No Ct	No Ct	No Ct
	before inoculation 1	No Ct		
	before inoculation 2	No Ct	No Ct	
	before inoculation 2	No Ct		
	before inoculation 3	No Ct	No Ct	
	before inoculation 3	No Ct		
Non-template	Non-template control 1	No Ct	No Ct	No Ct
	Non-template control 2	No Ct		
S-1 Control_4 dpi[c]	TSWV inoculation 1	23.12	23.50	27.02
	TSWV inoculation 1	23.88		
	TSWV inoculation 2	27.91	28.06	
	TSWV inoculation 2	28.21		
	TSWV inoculation 3	29.86	29.50	
	TSWV inoculation 3	29.14		
Sw-7_4 dpi	TSWV inoculation 1	27.33	27.22	27.43
	TSWV inoculation 1	27.10		
	TSWV inoculation 2	24.81	24.45	
	TSWV inoculation 2	24.45		
	TSWV inoculation 3	30.26	30.63	
	TSWV inoculation 3	31.00		
S-1 Control_7 dpi	TSWV inoculation 1	23.16	22.88	22.46
	TSWV inoculation 1	22.59		
	TSWV inoculation 2	18.10	18.10	
	TSWV inoculation 2	28.42		
	TSWV inoculation 3	25.89	26.40	
	TSWV inoculation 3	26.91		
Sw-7_7 dpi	TSWV inoculation 1	38.17	35.94	35.01
	TSWV inoculation 1	33.71		
	TSWV inoculation 2	34.74	34.63	
	TSWV inoculation 2	34.52		
	TSWV inoculation 3	34.31	34.47	

	TSWV inoculation 3	34.63		
S-1 Control_14 dpi	TSWV inoculation 1	16.35	16.35	22.54
	TSWV inoculation 1	16.36		
	TSWV inoculation 2	15.60	16.03	
	TSWV inoculation 2	16.45		
	TSWV inoculation 3	35.40	35.25	
	TSWV inoculation 3	35.09		
Sw-7_14 dpi	TSWV inoculation 1	32.61	29.41	33.04
	TSWV inoculation 1	26.20		
	TSWV inoculation 2	32.66	30.51	
	TSWV inoculation 2	28.35		
	TSWV inoculation 3	39.28	39.22	
	TSWV inoculation 3	39.15		
S-1 Control_21 dpi	TSWV inoculation 1	15.84	15.90	16.88
	TSWV inoculation 1	15.95		
	TSWV inoculation 2	16.74	16.43	
	TSWV inoculation 2	16.12		
	TSWV inoculation 3	15.20	18.32	
	TSWV inoculation 3	18.32		
Sw-7_21 dpi	TSWV inoculation 1	32.75	33.03	31.51
	TSWV inoculation 1	33.30		
	TSWV inoculation 2	30.76	27.49	
	TSWV inoculation 2	24.22		
	TSWV inoculation 3	No Ct	34.02	
	TSWV inoculation 3	34.02		
S-1 Control_35 dpi	TSWV inoculation 1	21.10	19.14	20.48
	TSWV inoculation 1	17.17		
	TSWV inoculation 2	21.09	18.46	
	TSWV inoculation 2	15.82		
	TSWV inoculation 3	24.89	23.85	
	TSWV inoculation 3	22.80		
Sw-7_35 dpi	TSWV inoculation 1	33.47	31.53	32.41
	TSWV inoculation 1	29.58		
	TSWV inoculation 2	34.03	34.01	
	TSWV inoculation 2	33.99		
	TSWV inoculation 3	33.75	31.71	
	TSWV inoculation 3	29.66		

[a]S-1 control is a TSWV susceptible breeding line, Fla. 8059 used as a recurrent parent for backcrossing.

[b]Sw-7 is near-isogenic line containing Sw-7 gene for TSWV resistance through backcrossing to the Fla. 8059 for BC6 generations, theoretically 98.438% identical to the recurrent parent Fla. 8059.

[c]dpi: days post inoculation with TSWV.

[d]Viral titer measurement in TSWV-inoculated S-line and *Sw-7* line was conducted using real time RT-PCR on leaf samples collected at 4, 7, 14, 21 and 35 dpi, with the corresponding Ct values presented.

Supplementary Table S2. Differentially expressed tomato genes to have intersected at all time points in Venn diagram

Gene accession	Gene annotation	0 dpi[a]	4 dpi	7 dpi	14 dpi	21 dpi	35 dpi
Solyc04g081610	Unknown Protein	11.665	11.000	11.354	10.792	10.309	10.907
Solyc04g081710	Unknown Protein	13.774	13.162	6.740	13.508	13.350	14.255
Solyc12g013530	Unknown Protein	-1.889	-2.837	-3.059	-3.837	-5.644	-4.644
Solyc12g013750	Mannan endo-1,4-beta-mannosidase 3	-6.644	-6.644	-5.767	-6.127	-6.727	-5.545
Solyc12g013960	Unknown Protein	-2.000	-2.837	-3.474	-3.644	-4.322	-3.644
Solyc12g014280	Chloroplastic group IIA intron splicing facilitator CRS1, chloroplastic	-2.059	-2.737	-3.059	-2.644	-2.943	-3.644
Solyc12g015630	Genomic DNA chromosome 5 P1 clone MDF20	-5.059	-3.837	-4.644	-5.059	-5.059	-5.059

[a]dpi: days post inoculation.

Supplementary Table S3. Selected differentially expressed photosynthetic pathway genes in *Sw-7* compared to the S-line after TSWV inoculation.

S. lycopersicum accession	Annotation	4 dpi[a]	7 dpi	14 dpi	21 dpi	35 dpi
Chlorophyll binding protein						
Solyc02g070950	Chlorophyll a/b binding protein	-	-	-	1.664	-
Solyc02g070970	Chlorophyll a/b binding protein	-	-	-	1.646	-
Solyc12g011450	Chlorophyll a-b binding protein 13	-	-	-	-	1.884
Solyc07g063600	Chlorophyll a-b binding protein 13	-	-	-	1.516	-
Solyc03g005780	Chlorophyll a-b binding protein 3C-like	-	-	-	1.669	-
Solyc10g006230	Chlorophyll a-b binding protein 7	-	-	-	1.411	1.888
Cytochrome complex						
Solyc01g007540	Cytochrome b6-f complex subunit 4	2.485	-	-	1.570	-
Photosystem subunit protein						
Solyc08g013670	Photosystem I reaction center subunit	-	-	-	-	1.824
Solyc12g044280	Photosystem I reaction center subunit	-	-	-	1.628	-
Solyc06g066640	Photosystem I reaction center subunit	-	-	-	1.511	-
Solyc08g006930	Photosystem I reaction center subunit X	-	-	-	1.536	-
Solyc01g008510	Photosystem II 5 kDa protein,	-	-	-	-3.474	-3.059
Solyc01g102770	Photosystem II reaction center protein Z	-	-	-	1.561	
Solyc06g084050	Photosystem II reaction center W	-	-	-	1.521	

[a]dpi: days post inoculation.

Supplementary Table S4. Evaluating transgenic plants expressing OLP-PR5 for their resistance to TSWV in comparison to that of the transgenic GFP and non-transgenic 'Moneymaker' plants

Treatment	Plant No.	7 dpi[a]	14 dpi	21 dpi	28 dpi	35 dpi	Infection status[b]
Transgenic GFP	1	30.66	29.25	19.10	19.33	24.38	(+)
	2	29.43	23.61	19.77	21.51	24.42	(+)
	3	No Ct	30.43	23.24	23.42	25.63	(+)
	4	34.45	No Ct	23.61	23.28	26.54	(+)
	5	34.00	20.16	20.41	20.27	22.97	(+)
Transgenic OLP-PR5	1	No Ct	34.25	No Ct	36.23	37.45	(-)
	2	36.98	36.88	No Ct	35.20	32.36	(-)
	3	No Ct	No Ct	36.11	21.32	23.63	(+)
	4	38.47	38.03	37.88	27.43	33.28	(-)
	5	37.55	22.14	20.81	22.02	24.93	(+)
Control tomato ‘Moneymaker’	1	29.18	17.59	20.02	22.62	20.60	(+)
	2	19.68	18.77	20.19	20.53	20.54	(+)
	3	22.27	18.26	21.11	22.36	21.57	(+)
	4	24.83	23.20	19.50	20.43	22.91	(+)
	5	25.53	25.39	24.09	24.33	22.75	(+)
Healthy control	1	No Ct	37.08	No Ct	37.22	36.69	(-)
Positive control	1	19.10	19.81	28.81	21.92	20.90	(+)
	2	26.49	27.09	25.65	25.64	20.90	(+)
Non-template control	NTC	35.51	No Ct	No Ct	37.31	35.64	(-)

[a]dpi: days post inoculation.

[b]Ct values >35.00 to No Ct were considered negative (-); Ct values <30 were considered positive (+); Ct valves 30-35 were considered border lines.

SUPPLEMENTARY FIGURES:

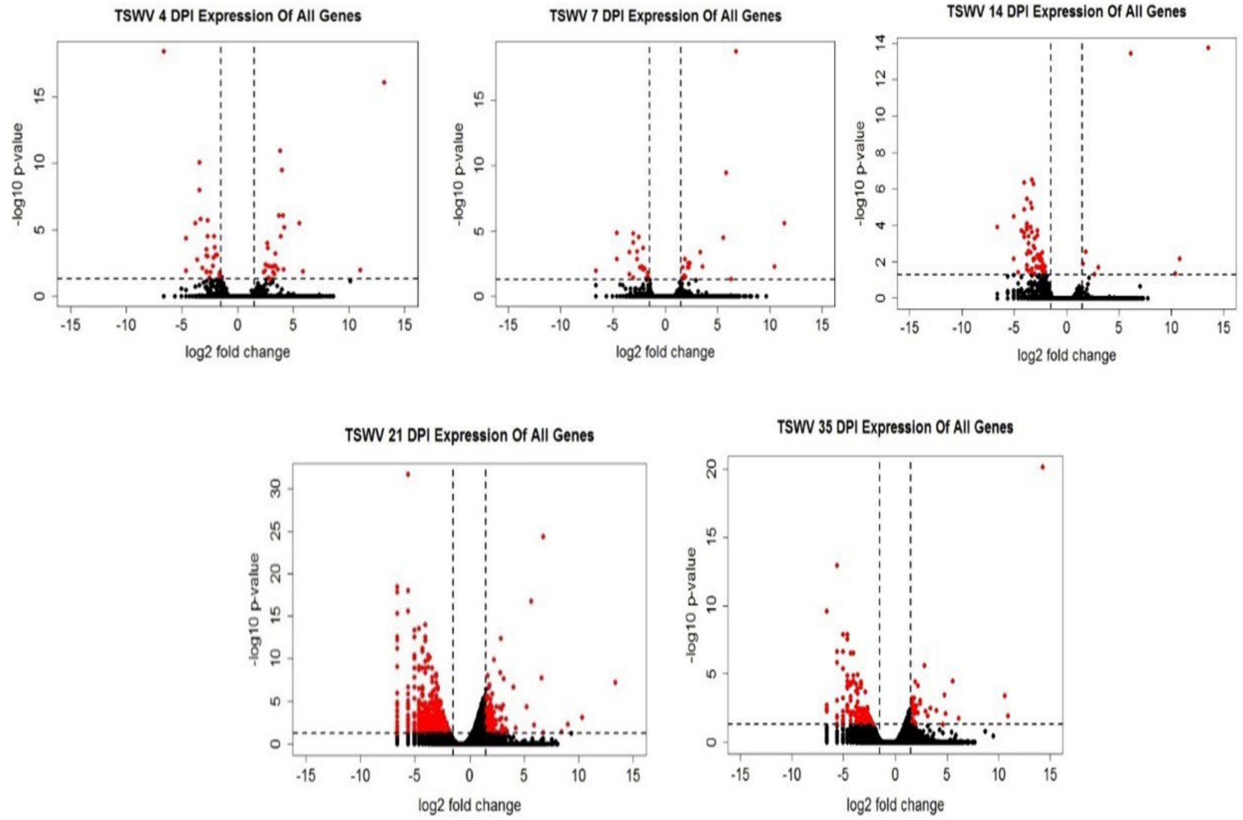

Supplementary Figure S1. The Volcano plots illustrate the distribution of all expressed genes and highlighted DEGs with the number of up- and down-regulated genes for each time point on 4, 7, 14, 21 and 35 dpi. Red dots on the right indicate *Sw-7* induced genes and red dots on the left indicate suppressed in *Sw-7*. X-axis represents -log10 (p-value) and y-axis represents log2 (fold change). Black horizontal dotted lines show the p-value cut off at 0.05. Black vertical dotted lines were drawn using log2 (fold change) cut off at -1.5 and 1.5 demonstrating suppressed and induced genes respectively in the *Sw-7* line.

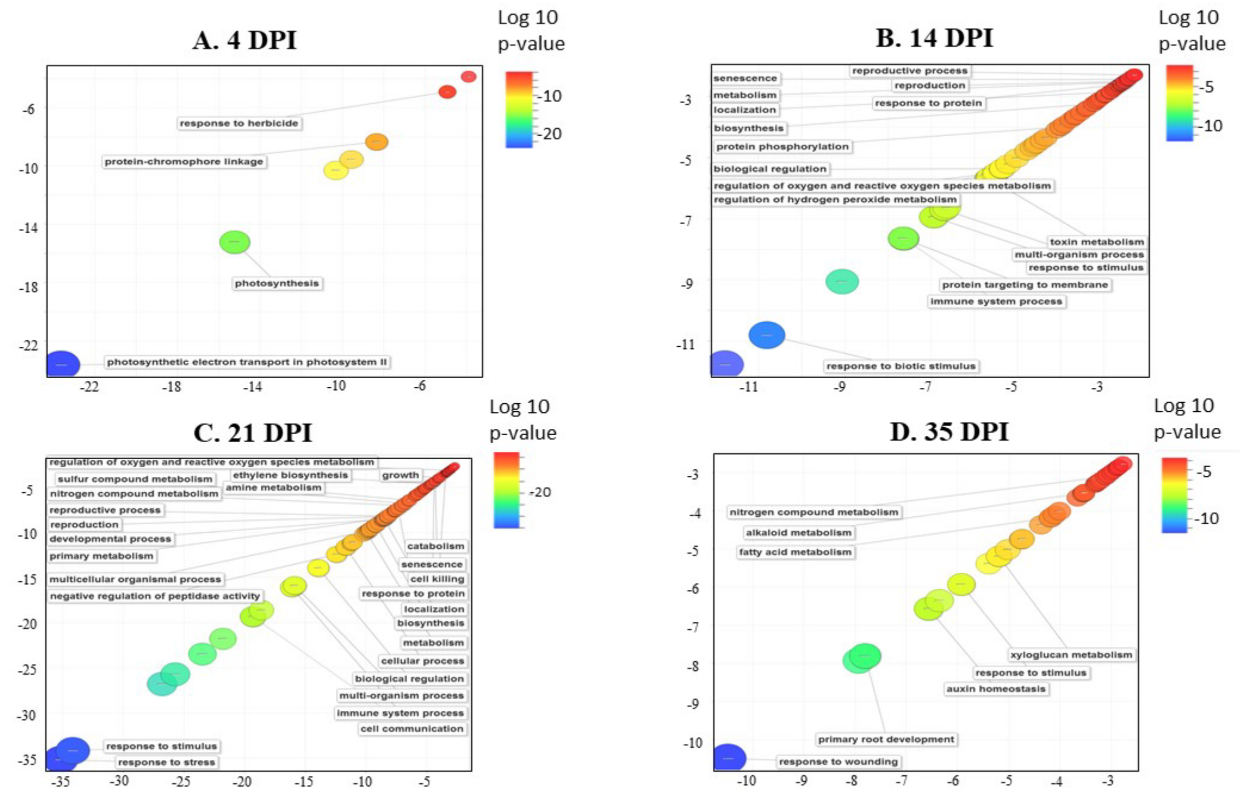

Supplementary Figure S2. Scatterplot showing cluster representatives of differentially expressed genes. Figures were generated with the REVIGO program using the list of enriched GO terms at each time point.

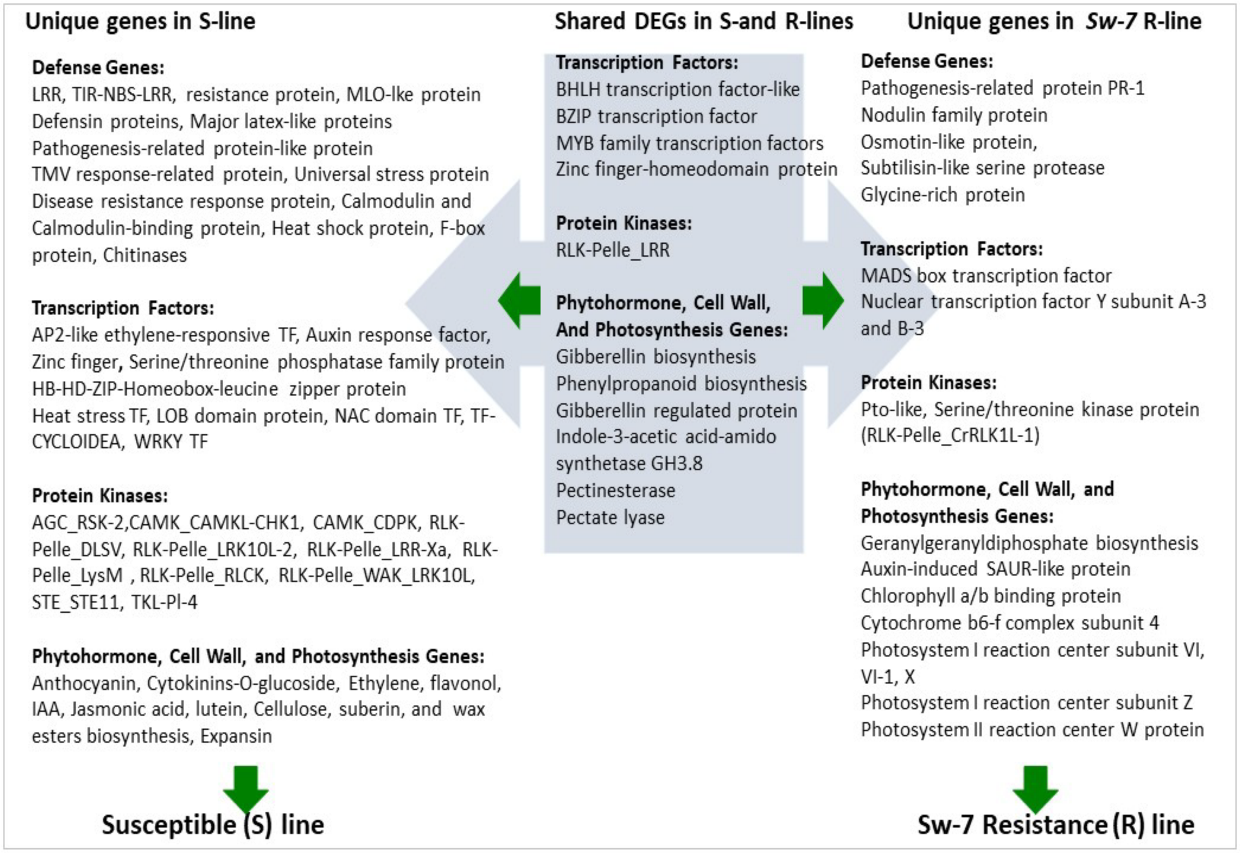

Supplementary Figure S3. Summary of the differentially expressed genes (DEGs). We have classified DEGs in four major groups: including a) Defense genes, b) Transcription factors, c) Protein kinases, and d) Phytohormones, cell wall and photosynthesis genes. In each category, certain group of genes are induced in the TSWV-resistant (*Sw-7*) plants (right panel), or in the susceptible (*S*) line (left panel). We have placed a small group of gene members, expressed in both the resistance (*Sw-7*) and the susceptible line in the middle panel.
